# Glycaemic Effects of Non-statin Lipid-Lowering Therapies

**DOI:** 10.1007/s11886-016-0795-9

**Published:** 2016-11-19

**Authors:** Patrick D. Collins, Naveed Sattar

**Affiliations:** 1 University Hospital Lewisham, Lewisham High Street, London, SE13 6LH UK; 2 Institute of Cardiovascular & Medical Sciences, BHF Glasgow Cardiovascular Research Centre, University Of Glasgow, 126 University Place, Glasgow, G12 8TA UK

**Keywords:** Statins, Hypercholesterolaemia, PCSK9, Ezetimibe, Niacin, Diabetes

## Abstract

Since the publication of the JUPITER trial, attention has been focused on the adverse glycemic effects of statin therapy. Although the modest increase in the risk of new diabetes mellitus is outweighed by the reduction in cardiovascular events for statins, emerging biochemical and genetic links between lipid metabolism and glycemic control raise the prospect of a broader diabetogenic effect of lipid-lowering therapies. For the novel and powerful PCSK9-inhibitor class available evidence does not support a major glycaemic effect with the results of large scale trials awaited although preliminary genetic data does suggest a link. In contrast, there is clear evidence of a diabetogenic effect for the now outdated but well-studied niacin. For ezetimibe and fibrates, evidence is scarce but currently broadly unconcerning. For now, the glycemic effects of lipid-lowering therapies should have a limited influence on clinical decision-making. Further study in this topical area is needed.

## Introduction

Hypercholesterolaemia is one of the most important modifiable risk factors for cardiovascular disease. Efficacious for primary prevention, even in individuals with only modest cardiovascular risk, statins are some of the most widely used medicines worldwide. Despite this, a very small proportion of individuals do not sufficiently respond or are unable to tolerate these drugs. Although the risks of liver derangement, myositis, and even rhabdomyolysis, have long been recognised, it was not until the publication of the 2008 randomised JUPITER trial that focus turned to modest but significant increases in the new development of diabetes mellitus [1]. Individuals receiving maximal doses of rosuvastatin had a 28 % excess rate of diabetes (mainly confined to those with preexisting risk factors) compared to the placebo arm. Subsequent randomised trial meta-analyses showed a 9–11 % increased risk of diabetes amongst statin users [2 3•] with evidence of a higher rates amongst those on intensive statin therapy [4].

In vitro and population studies have revealed possible mechanistic explanations. Firstly, ‘off-target’ effects potentiating insulin resistance (inhibition of GLUT4 translocation to the cell membrane and of adipocyte differentiation) or decreased insulin secretion (inhibiting calcium uptake into pancreatic beta cells and induction of beta cell apoptosis) have been proposed [5]. It is suggested that these effects are concentrated amongst the lipophilic statins—which may account for the reduced risk of new-onset diabetes originally seen amongst users of the hydrophilic pravastatin in the pivotal West of Scotland Coronary Prevention Study [6]. However, this line of argument remains speculative and diabetes risk was clearly elevated in the pravastatin arm of the PROSPER trial [7]. Secondly, there appear to be downstream ‘on-target’ effects of HMG-CoA reductase inhibition which alter insulin metabolism and weight and parallel clinical evidence that HMG-CoA reductase inhibition leads to minor weight gain amongst those who take statins [4]. In other words, it appears that at least part of the diabetogenic potential of statins is mediated via weight change although the precise mechanisms are yet to be elucidated but speculatively could include a a very small effect on exercise capacity sufficient to tip the balance towards very minor weight gain in some.

Finally, and perhaps most interestingly, there is emerging evidence of an association between alleles influencing lipid metabolism and the risk of diabetes mellitus. At one extreme, individuals with familial hypercholesterolaemia have a lower incidence of diabetes mellitus compared to unaffected family members—despite early and aggressive treatment with statins [8]. Broader mendelian randomisation studies (the use of genetic polymorphisms to help dissect causality) including tens of thousands of individuals have suggested an inverse relationship between single nucleotide polymorphisms (SNPs) predisposing to elevated LDL cholesterol and the risk of developing diabetes [9•], with particular alleles for the biological target of statin therapy implicated [4]. Whilst a number of potential confounding factors prevent firm conclusions (including survivor bias, pleiotropy, the greater use of statin therapy amongst individuals with high LDL), the foregoing observations raise the possibility of an inherent diabetogenic effect of lipid lowering therapies, especially LDL cholesterol-lowering therapies.

In this review, we summarise the evidence regarding the glycemic effects of non-statin lipid-lowering therapies, with results summarised (Table 1).

**Table 1:** Levels of evidence linking statins and non-statin-based lipid-lowering medications to diabetes risks plus any clinical ramifications

	Trial evidence	Genetic evidence	Clinical ramifications
Statins	Meta-analyses of multiple randomised trials confirm modestly elevated (9–11 %) diabetes risk and slight weight gain	HMG-CoA reductase SNPs associated with a small but discernible increase in the risk of type 2 diabetes, raised ﻿blood﻿ glucose and insulin, and higher body weight	Modest diabetes risks do not alter statin prescription recommendations to those with established disease or at elevated risk. However, the risk should be mentioned to patients to further incentivise lifestyle changes. HbA1c or fasting glucose should be checked prior to statin commencement.
PCSK9 inhibitors	Short-term (6–18 months) trial with alirocumab does not support a measurable effect on diabetes risk Longer term data needed, as well as data for other PCSK9 inhibitors	Emerging genetic evidence of slight increased (19%: 95% CI 2 to 38%) diabetes risk for genetically determined 1 mmol/l LDL-c reduction .	None as yet
Ezetimibe	Non-significant 9 % increased risk in IMPROVE-IT reported as abstract—yet to be fully published	Recent genetic evidence for a significant diabetes risk (Odds ratio 2.42; 95% CI 1.70 to 3.43) associated with alleles in or near NPC1L1, the molecular target for Ezetimibe.	None as yet
Niacin	34 % increased risk of new diabetes associated with the use of niacin in meta-analysis of trial data	No published data	None since niacin is no longer recommended due to its lack of clinical outcome benefit.
Fibrates	Prior limited evidence suggesting bezafibrate may lower diabetes risk but no clear evidence of an effect of fenofibrate in the FIELD trial	Mixed genetic data on the association of PPAR-alpha SNPs with diabetes: more studies needed	None

### PCSK9 Inhibitors

The PCSK9 inhibitors (including alirocumab, evolocumab and bococizumab, although the latter now being withdrawn) are an emerging drug class of monoclonal antibodies against PCSK9—a binding protein potentiating the degradation of hepatocyte LDL-R, which can be delivered subcutaneously at monthly or bimonthly intervals and offer potent reductions in LDL cholesterol (up to 61 %). Colhoun et al. examined the influence of alirocumab on transition to diabetes amongst 10 randomised control trials (*n* = 3448) with a high proportion of prediabetic subjects (39.6 %). They found no clear evidence of increased development of diabetes compared to placebo (HR 0.64 (0.36–1.14)) or ezetimibe (HR 0.55 (0.22–1.41)) [10••]. Furthermore, there was no significant change in HbA1c over time. However, the follow-up period was relatively short (6–18 months), and the total number of new cases of diabetes was limited (*n* = 218). Similarly, there was no significant glycemic effect of monthly evolocumab over a 52-week period in a randomised comparison with placebo (*n* = 901) [11] nor any glycemic effect amongst participants in a randomised trial against standard care who had preexisting dysglycaemia or metabolic syndrome over 52 weeks [12]. In a recent meta-analysis, we did not find any significant difference in lipid effects of evolocumab on diabetic patients versus controls [13]. Further work on the glycaemic effects of evolocumab is currently ongoing.

Although the data for PCSK9 inhibitors is thus far reassuring, a more subtle effect cannot be excluded and it is notable that widespread acceptance of the link between statins and diabetes came only after large scale meta-analysis including thousands of new cases of diabetes. Furthermore, most trials have examined the addition of PCSK9 inhibitors to statins which may partially mask a diabetogenic effect if mechanisms are shared between classes. Further work in this area is proceeding rapidly especially regarding SNPs related to PCSK9 function and glycaemic control as well as to weight changes. Such work requires the integration of results from multiple large studies to yield sufficient power and is now readily achievable in an era of both major genetic studies and distributed information technology. Indeed, a recent relevant study exploiting genetic data has examined this issue and reported genetic variants in PCSK9 were associated with a 19 % (95 % CI 2 to 38 %) higher risk for diabetes per mmol/l lower LDL-c levels [14••]. This is the first large genetic study linking PCSK9 snps to diabetes risk and reports a significant higher risk, albeit modestly so, but with stronger associations with diabetes for genes encoding the molecular targets for ezetimibe. Nevertheless, the results of ongoing cardiovascular end-point trials are needed to determine the true effect of short term PCSK9 inhibition on diabetes risks. Fortunately, we will not have to wait too long for these to report and all have pre-specified assessment of diabetes risk and HbA1c from the outset.

One final comment deserves mention. Since these drugs are currently being targeted to those at very high risk of cardiovascular events—i.e. patients with either familial hypercholesterolaemia or preexisting cardiovascular disease with uncontrolled LDL cholesterol despite maximally tolerated statin and/or ezetimibe therapy—it is unlikely that a modest diabetogenic effect would alter treatment decisions [15•].

### Niacin

The B vitamin niacin initially showed promise, with a reduction in 17 % reduction in MI seen in results from the Coronary Drug Project [16], but despite beneficial effects on serum LDL no clear benefit in cardiovascular events or mortality was seen in addition to statin therapy [17, 18]. As well as the well-recognised flushing effects of niacin, one notable side effect is a worsening of glycemic control. Although early work concluded this effect was modest and unlikely to lead to the onset of diabetes [19], post hoc analysis, new trials and a well conducted meta-analysis of both published and unpublished work are concordant in suggesting a significant effect. Over a 5-year follow-up, in the Coronary Drug Project’s randomised comparison of niacin to placebo, patients with impaired fasting glucose tolerance at baseline progressed to diabetes at a rate of 19.8 % on niacin versus 15.2 % on placebo (HR 1.34 (1.00–1.80)) with a similar (although non-significant) relative risk increase amongst those with normal glucose tolerance (HR 1.41 (0.97, 2.05)) [20]. In the HPS2-THRIVE comparison of the addition of niacin or placebo to those already on statins there was a 55 % increase in serious disturbances in glycemic control (for which many patients were hospitalised), affecting 11.2 % of diabetics assigned to niacin [7]. Furthermore, there was a 30 % increase in the risk of new-onset diabetes in the treatment group. Goldie et al. performed a rigorous meta-analysis on the effect of niacin on the risk of new-onset diabetes [21•]. Overall, they found a 34 % increased risk of new diabetes associated with the use of niacin (although this may be a slight overestimate as trials with no new cases of diabetes were excluded). Niacin is no longer in widespread clinical use but these results do add to concerns of an implicit diabetogenic effect of lipid-lowering therapies.

### Ezetimibe

Ezetimibe inhibits enterocyte absorption and increases hepatocyte uptake of cholesterol and currently is the mainstay of therapy in individuals who do not tolerate statins or require further LDL cholesterol reductions. Added to statin therapy, it offers both a 15 % absolute reduction in LDL cholesterol [22] and a modest reduction in cardiovascular events over a 7-year post-acute coronary syndrome ((HR 0.94 (0.89, 0.99)) [23], but data regarding its glycemic effects are rather limited. Saito et al. randomised diabetics to ezetimibe or placebo and did not find any significant effect on glycemic control (assessed by either HbA1c or fasting plasma glucose) or exacerbations of diabetes over a short follow-up period of 24 weeks [24]. However, 9 % of diabetics in the ezetimibe group had their drug regime altered due to hyperglycaemia versus 4 % of the placebo group. Although the trial was well conducted, its very limited sample size (*n* = 152), single nationality population (Japanese) and exclusion of those with poor diabetic control, preexisting cardiovascular disease or concurrent statin use means wider interpretation of its results is fraught. In the IMPROVE-IT randomised comparison of additional ezetimibe or placebo for subjects on statins with recent acute coronary syndrome a preliminary report, not yet peer-reviewed, suggests ezetimibe resulted in a small, non-statistically significant, increase in the relative risk of new-onset diabetes (9 % per 1 mmol/L reduction in LDL cholesterol) [25•]. These clinical data should be contrasted with recent genetic data [14••] which suggests a much larger effect on diabetes risk of lifelong inhibition of the genes encoding the molecular targets of ezetimibe: a 2.42 (95 % CI 1.70 to 3.43) odds ratio per mmol/l lower LDL-c. Therefore, longer term data on diabetes risks with ezetimibe treatment are needed to rule out a diabetes risk.

### Fibrates

Fibrates interact with peroxisome proliferator-activated receptors to reduce plasma LDL cholesterol and triglycerides. Although they reduce the rate of cardiovascular events, they do not affect all-cause mortality or rates of cardiovascular death [26]. Overall there is very limited data on the interaction between fibrates and glycemic control. The FIELD study randomised diabetic subjects not on any statin therapy at baseline (*n* = 9795) to either fenofibrate or placebo. Over 6 years, the change in HbA1c was negligible regardless of randomisation but formal statistical testing was not reported [27]. Lee et al. examined the effects of fibrates on the rate of new-onset diabetes amongst Taiwanese patients aged 55–59 with dyslipidaemia (*n* = 3815) over a 5-year period [28]. Although the overall rate of new-onset diabetes was unconcerning (3.8 %), the number of new diabetes cases was limited (*n* = 145) and the lack of a comparison group means no firm conclusions can be drawn. Tennabaum et al. found a reduced rate of diabetes over a 6-year follow-up amongst subjects with impaired fasting glucose and known coronary artery disease assigned to benzafibrate rather than placebo [29]. There are also mixed data on the association of PPAR-alpha SNPs and diabetes risk, and thus, overall, no clear conclusions can yet be drawn [30]. Newer classes of fibrate drugs are currently being tested with respect to cardiovascular outcome risks and it is likely that such studies will also include measures of glycaemia as pre-specified outcomes.

## Conclusions

It is striking that both genetic studies and large scale meta-analysis for statins and niacin (the two lipid-lowering therapies for which there is a reasonable body of data) point towards an inverse link between LDL cholesterol and glycemic control, although niacin also influences HDL-cholesterol levels which may have separate links to diabetes. However, it is not yet clear that all lipid-lowering therapies are implicitly diabetogenic, or that all statins are equally diabetogenic [31]).

There are a number of gaps in the literature. Further studies delineating the mechanisms by which statins cause diabetes are clearly needed. Both ezetimibe and PCSK9 inhibitors lack sufficient clinical trial data to exclude a diabetogenic effect, although, as noted, large scale trials of PCSK9 inhibitors are underway, and will also examine diabetes outcomes, important given emerging evidence of a link between PCSK9 genetic variants and diabetes risk.

For PCSK9 inhibitors, any diabetogenic effect would have to be very significant to further limit their use beyond individuals at high risk of cardiovascular events without other proven options. For statins, the benefits clearly outweigh the risks, even for primary prevention. Potential diabetogenic effects can be easily monitored, managed and mitigated by lifestyle changes. Indeed, a positive consequence of this area is that more patients at risk of, or with existing cardiovascular disease, are screened for diabetes, receive preventative advice and have earlier diagnosis and treatment.
